# Bio-based anode material production for lithium–ion batteries through catalytic graphitization of biochar: the deployment of hybrid catalysts

**DOI:** 10.1038/s41598-024-54509-8

**Published:** 2024-02-17

**Authors:** Ziyi Shi, Yanghao Jin, Tong Han, Hanmin Yang, Ritambhara Gond, Yaprak Subasi, Habtom Desta Asfaw, Reza Younesi, Pär G. Jönsson, Weihong Yang

**Affiliations:** 1 Department of Material Science and Engineering, KTH Royal Institute of Technology, 114 28 Stockholm, Sweden; 2 Department of Chemistry-Ångström Laboratory, Uppsala University, Lägerhyddsvägen 1, Box 538, 75121 Uppsala, Sweden

**Keywords:** Biochar, Pyrolysis, Catalytic graphitization, Bio-graphite, Lithium-ion battery, Batteries, Materials science, Batteries

## Abstract

Producing sustainable anode materials for lithium-ion batteries (LIBs) through catalytic graphitization of renewable biomass has gained significant attention. However, the technology is in its early stages due to the bio-graphite's comparatively low electrochemical performance in LIBs. This study aims to develop a process for producing LIB anode materials using a hybrid catalyst to enhance battery performance, along with readily available market biochar as the raw material. Results indicate that a trimetallic hybrid catalyst (Ni, Fe, and Mn in a 1:1:1 ratio) is superior to single or bimetallic catalysts in converting biochar to bio-graphite. The bio-graphite produced under this catalyst exhibits an 89.28% degree of graphitization and a 73.95% conversion rate. High-resolution transmission electron microscopy (HRTEM) reveals the dissolution–precipitation mechanism involved in catalytic graphitization. Electrochemical performance evaluation showed that the trimetallic hybrid catalyst yielded bio-graphite with better electrochemical performances than those obtained through single or bimetallic hybrid catalysts, including a good reversible capacity of about 293 mAh g^−1^ at a current density of 20 mA/g and a stable cycle performance with a capacity retention of over 98% after 100 cycles. This study proves the synergistic efficacy of different metals in catalytic graphitization, impacting both graphite crystalline structure and electrochemical performance.

## Introduction

Lithium-ion batteries (LIBs) are extensively used in various applications from portable electronics to electric vehicles (EVs), and to some extent in stationary energy storage systems^1–4^. The negative electrodes in most commercial LIBs contain graphite because of its low de-/lithiation potential (0 to 250 mV vs Li^+^/Li) and high practical gravimetric capacity of 300 to 360 mAh g^−1^ (the theoretical capacity is 372 mAh g^−1^)^5–9^.

Owing to the extension of EVs market, the demand for lithium-ion cells and batteries is expected to keep rising. By 2030, the global LIBs capacity is predicted to exceed 3000 GWh, which is over 400% of the capacity in 2022 (700 GWh)^10,11^, which means there will be an increased demand for graphite in the future^12,13^. Both natural and synthetic (or artificial) graphite used in batteries originate from fossil-based resources which are not sustainable^14–17^. As of 2014, natural graphite has been listed by the EU as one of the critical raw materials considered of high economic importance but subject to supply risk^18^. On top of the supply issue, it is worth noting that mining, purification and beneficiation of natural graphite can be energy-intensive, produce greenhouse gases, generate mining dust, and require using chemicals like HF which are toxic and pose considerable safety risk to humans, the environment (soil, air, and water), and aquatic life^14,15,19^. Synthetic graphite is commonly obtained by carbonizing fossil-based precursors such as coal, coal tar, pitch or petroleum coke at temperatures as high as 3000 °C, which demands significant amount of energy and results in non-combustion greenhouse emissions (from the process and the preparation of fossil-based carbon precursor)^16,17,20^.

To address the challenges mentioned above, there has been a lot of effort to produce graphite materials by using transition metals^21,22^. Biomass can be an ideal candidate as a carbon precursor because of its renewability, sustainability, abundance and carbon–neutral nature^23,24^. Fromm et al. carried out experiments using various biomass sources such as birch, oak, and bamboo wood to produce graphite materials without a catalyst^25^. They varied the temperature between 800 and 2800 °C and found that biomass-derived carbon cannot be directly converted into graphitic carbon even at high temperatures. A. Oya et al. introduced metallic elements as catalysts in graphitization and converted non-graphitizable materials (resin) into graphitic materials with good graphite crystalline^26,27^, which verified the feasibility of catalytic graphitization. Hoekstra et al. discussed the active temperatures of different base metal catalysts (copper, nickel, cobalt, and iron salts) in graphitization and reported that iron, nickel and cobalt are effective catalyst at the lowest temperature (temperature over 715 °C), whereas the nickel and cobalt nanoparticles were only activated in catalytic graphitization at a temperature over 800 °C^28^. Afterward, E. Thompson et al. developed a production process of graphitic carbon by heating a mixture of softwood sawdust and iron nitrate, which provided a promising route to the large-scale and sustainable synthesis of graphite for electrode applications^29^. Previous studies on catalytic graphitization directly utilized biomass as the carbon precursor, which typically contains abundant volatiles and relatively low fixed carbon. Comparatively, biochar derived through pyrolysis has over 80% fixed carbon, making it favorable for catalytic graphitization. Nonetheless, there are only a few studies using biochar as a carbon precursor for graphite synthesis.

On the other hand, it is worth noting that the synthetic graphite materials in literature exhibited lower graphitization degrees or subpar electrochemical performances compared to commercial graphite. Some research was done to further improve the graphitic crystalline of bio-based synthetic graphite materials, with a particular focus on the modification of catalysts. Major et al. developed a bimetallic hybrid catalyst (iron and cobalt) and compared it with a single-metal catalyst^30^. Their results concluded that the sample treated with the hybrid catalyst performed a better graphitic structure after graphitization. Kamal et. al reported a catalytic graphitization experiment based on a hybrid catalyst consisting of a non-metallic element (silica) and a metallic element (iron)^31^. This study found that combining different elements could generate multi-element complexes that performed a new catalytic activity. However, there is a lack of study on the influence of trimetallic hybrid catalysts on graphitization and the electrochemical characterization of the bio-based synthetic graphite.

In this work, catalytic graphitization of biochar over hybrid catalysts was studied. Specifically, biochar pellets derived via pyrolysis of sawdust at 550 °C were used as carbon precursors for the graphite synthesis. Bimetallic hybrid catalysts (Ni and Fe with ratios of 1:1, 1:2, and 2:1) and a trimetallic hybrid catalyst (Ni, Fe, and Mn with a ratio of 1:1:1) were deployed in the graphitization process. Additionally, a single-metal catalyst (Ni) was also employed as a reference as it has been recognized as an effective metal catalyst for graphitization at the temperature over than 800 °C^28^. The synthetic bio-graphite products derived from the three types of catalysts were evaluated from the perspective of the physical structure (the degree of graphitization, BET surface area and morphology) and electrochemical performance in a half-cell (reversible capacity and long-term cycling). The overall objective of the study is to screen out a more efficient catalyst for graphitization through a comprehensive analysis of graphite samples and explore the possibility of utilizing biochar as a carbon precursor for the production of bio-based synthetic graphite (bio-graphite) for LIBs.

## Method

### Materials

Raw biochar pellets provided by Envigas AB were used as carbon precursors for catalytic graphitization. The biochar pellets were initially obtained from the pyrolysis of sawdust at 550 °C by using an auger reactor system. The proximate analysis and elemental analysis results were provided by Eurofins AB, as shown in Table 1. Fe(NO_3_)_3_, Ni(NO_3_)_2_, and Mn(NO_3_)_2_ with reagent grades were purchased from Sigma-Aldrich and used as catalysts in this work. Hydrochloric acid (1 mol/L, ACS reagent) purchased from Sigma-Aldrich was used for ash leaching.

**Table 1:** Ultimate and proximate analysis of the biochar used for catalytic graphitization.

Elemental analysis (wt%, dry basis)	
C	83.50 ± 3.41
H	2.68 ± 0.24
N	0.26 ± 0.05
S	0.21 ± 0.04
Cl	–
O^a^	13.35
Proximate analysis (wt%)	
Moisture (as received)	2.71 ± 0.43
Volatile (dry basis)	13.71 ± 0.16
Ash (dry basis)	3.33 ± 0.09
Fixed carbon^a^ (dry basis)	82.96

^a^Calculated from difference: O% = 100%-C%-H%-N%-S%. Fixed carbon% = 100%-Volatile%-Ash%

### Synthesis of graphite

**Figure 1:**
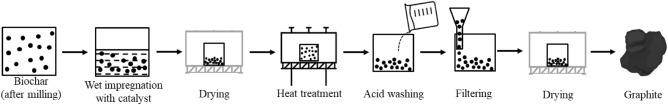
Illustration of the bio-graphite synthesis from catalytic graphitization.

As mentioned before, the effects of three different types of catalysts were investigated systematically in this study. Figure 1 illustrates the synthesis method used in this work. Before the experiment, the carbon precursors, biochar pellets, were loaded into a zirconia jar with zirconia balls and crushed by using a planetary ball mill (QM-3SP2). The number of zirconia balls added in the jar was 20, and the ball-to-powder mass ratio was 10:1. After milling and sieving, only biochar particles smaller than 32 µm were used as feedstock for graphitization. For each test, 20 g of biochar powders were loaded. The loading of the catalyst was defined as the mass ratio of metal content in the catalyst to biochar. To mix catalyst and biochar, a certain amount of catalyst was first dissolved in 100 ml of deionized water. The biochar sample was subsequently mixed with the prepared solutions followed by magnetic stirring (5 h) and drying (24 h) in air at 100 °C. Then the mixture of biochar and catalyst was loaded into an alumina crucible and then placed into a horizontal tube furnace for heating at a ramp of 20 °C/min in a 200 ml/min of N_2_ gas flow to 1300 °C and maintained for 3 h. Detailed parameters of the cases, including the catalyst composition, catalyst loading ratios, and corresponding definition of the produced graphite samples, are compiled in Table 2.

**Table 2:** Detailed descriptions of catalytic graphitization cases.

Name	Catalyst composition	Loading ratios
G-Ni	Ni(NO_3_)_2_	–
G-FeNi-11	Fe(NO_3_)_3_ and Ni(NO_3_)_2_	1:1
G-FeNi-12	Fe(NO_3_)_3_ and Ni(NO_3_)_2_	1:2
G-FeNi-21	Fe(NO_3_)_3_ and Ni(NO_3_)_2_	2:1
G-FeNiMn-111	Fe(NO_3_)_3_, Ni(NO_3_)_2_ and Mn(NO_3_)_2_	1:1:1

Thereafter, the metal catalysts in the graphite samples were removed via acid leaching. The impure graphite was soaked in 1 mol/L hydrochloric acid (HCl), filtered, and washed with deionized H_2_O. Specifically, the impure sample was first loaded into a beaker with 0.6 L of a HCl solution. A magnetic stirring heater was used to heat and stir the raw product for 1 h. Thereafter, the graphite powder was filtered away from the waste liquid. After separating the powders and waste liquid, NaOH was ejected into the waste liquid to determine the existence of metal ions in the liquid. The ash leaching was repeated until no solid was observed after ejection. Thereafter, the graphite powder was further washed with deionized water until a neutral pH value was obtained. Finally, the wet graphite powder product with a high purity was dried in a drying oven at 120 °C for 6 h.

### Characterization of the synthetic bio-graphite samples

Powder X-ray diffraction (XRD) patterns of the biochar and samples were obtained using a Bruker D8 Twin-Twin diffractometer equipped with Cu Kα X-ray radiation at 40 kV and 15 mA. The instrument was operated by scanning the Bragg angle (2θ) from 5° to 70° with a step size of 0.02°/s. The interlayer spacing of each sample was calculated from the Bragg angle of the diffraction peak for the 002 plane in the XRD spectra using Bragg's equation^32,33^:

1 $$\begin{array}{c}{d}_{002}=\frac{\lambda }{2sin\theta } \end{array}$$

where λ represents the X-ray wavelength of Cu Kα (λ = 0.15406 nm). θ represents the Bragg angle. d_002_ is the interlayer spacing of the carbo samples.

After the calculation of the interlayer spacing, the degree of graphitization (G%) could be determined as follows^34^:

2 $$\begin{array}{c}G\%=\frac{0.3440-{d}_{002}}{0.3440-0.3354}*100.\end{array}$$

where 0.344 is the d spacing of completely non-graphitized carbon; and 0.3354 is the d spacing of ideal graphite crystals.

The stacking height (*L*_c_-002 plane) and the crystallite size (*L*_a_-100 plane) of each sample were calculated using the following equation^33,35^:

3 $$\begin{array}{c}{L}_{a}=\frac{{k}_{1}\lambda }{{\beta }_{(100)}cos\theta }\end{array}$$

4 $$\begin{array}{c}{L}_{c}=\frac{{k}_{2}\lambda }{{\beta }_{(002)}cos\theta }\end{array}$$

where k is the Scherrer parameter (k_1_ = 1.84, k_2_ = 0.94), β represents the full width at half maximum (FWHM) of the diffraction peak.

Scanning electron microscopy (SEM) was conducted to determine the morphological characteristics by using a S4800 Hitachi SEM system (20 kV and 10 mm working distance) with a back scattered electron (BSE) signal. The energy-dispersive X-ray spectroscopy (EDS) detector from Oxford Instruments was further equipped on the SEM system to perform elemental composition and surface mapping determination to the graphite samples.

The surface area and pore structure of the biochar and the engineered catalysts were determined by means of N_2_ adsorption–desorption isotherms operated at 77 K using a Micromeritics ASAP 2000 instrument. The surface area was calculated by using the Brunauer–Emmett–Teller (BET) equation.

Raman spectra were acquired with a Tyrode I Raman microscope equipped with a 532-nm wavelength diode laser. The conversion degree (α) from a disordered carbon to a graphitic carbon was calculated as follows:

5 $$\begin{array}{c}\alpha =\frac{{I}_{G}}{{I}_{G}+{I}_{D}}*100.\end{array}$$

where I_G_ and I_D_ represent the intensities of the G and D bands in Raman spectra.

Thermogravimetric analysis (TGA) was performed on a Mettler TGA/DSC 3+ Stare system, equipped with a Huber minichiller 600 cooler. The samples were weighed into 100 µL Aluminum cups with pierced lid (the hole had approx. 0.4 mm diameter), and dry oxygen gas was flushed during the analysis. The samples were scanned from 25 to 900 °C, with a scan rate of 10 °C/min.

High-resolution transmission electron microscopy (HRTEM) was used to study morphologies and compositions with a JEOL JEM-2100F microscope operated at 200 kV.

### Electrode preparation and cycling

The electrochemical lithium (de)intercalation properties were examined with the coin-type (CR2032) half-cell setup. Composite carbon electrodes were prepared with a composition of 95 wt% synthetic bio-graphite as active material and 5 wt% CMC (sodium salt of carboxymethyl cellulose) as the binder. The mixture was ground to fine particles and blended together in water where 3 mL of deionized water was taken for 1 g of total weight. To ensure a homogeneous dispersion, the electrode paste was later completely shaken by using a high-energy T25 Ultra Turrax instrument (1 h, 10,000 rpm). Then, the paste was cast on a Cu foil and dried at ambient temperature for 30 min then punched into circular disks (diameter = 13 mm). Afterward, the electrodes (approximately 3–6 mg/cm^2^ of mass loading) were then transferred inside an MBraun Labstar glovebox maintaining an argon ambiance (H_2_O/O_2_ level < 0.5 ppm) and dried at 120 °C overnight in a Buchi oven inside the Glove box. The commercially available Li metal foil (Sigma Aldrich) was made into 15 mm circular disks as the counter electrode. LP57 (commercial grade) was chosen as an electrolyte. Two sheets of solupor were used as a separator for the counter and drenched with 100 uL of electrolyte. For electrochemical evaluation of graphitized carbon materials as anodes for LIBs, constant-current (galvanostatic) charge/discharge experiments were carried out over a potential range between 0.001 and 3.0 V versus Li/Li^+^ at 20 mA/g, in CCCV mode, at room temperature (25 °C). The experiments were performed by using a Biologic MPG2 potentiostat system. Potentiostatic Electrochemical Impedance Spectroscopy (PEIS) measurements were carried out right after 12 h resting or at Open Circuit Voltage (OCV) state followed by after 1st cycle, which is later compared with after 3 cycles at the same current, with a voltage amplitude of 5 mVrms between the frequency range of 100 kHz and 0.1 Hz. Galvanostatic discharge–charge at 0.1C was applied to reach the cutoff voltages (0.001–2.5 V), prior to the PEIS test, an OCV period of 1 h was imposed to allow cell relaxation.

## Results

### XRD

The XRD pattern of the raw biochar and the synthetic bio-graphite prepared using different catalysts at 1300 °C are shown in Figs. S1 and 2, respectively. The XRD pattern of the biochar included two broad peaks at around 25 and 44°, representing the (002) plane of the graphite sheet and the (100) plane of some aromatic structures, which indicated the presence of non-graphitic structure in the biochar^36,37^. After graphitization, the XRD spectrum displayed a distinctly different shape containing higher and sharper peaks at (002) and (100), indicating the effective catalytic behavior in carbon precursor. Two new strong diffraction peaks of (004) and (101) were observed for the synthetic bio-graphite. These resembled the natural graphite and sponge coke-based graphite, suggesting the formation of a typical graphitic crystalline structure. Among the catalytic graphitized samples, the G-FeNiMn-111 sample had the highest (002) peak, demonstrating that deploying the trimetallic hybrid catalyst could effectively improve the crystal structure of synthetic graphite.

**Figure 2:**
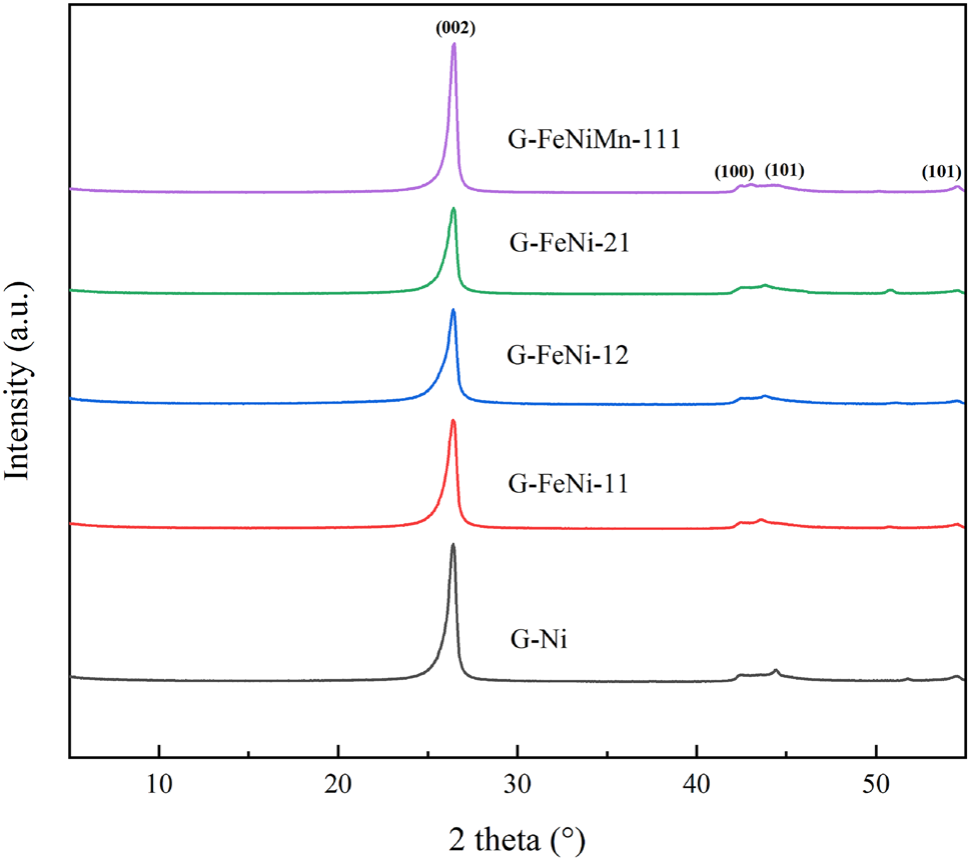
XRD patterns of raw biochar and synthetic bio-graphite samples (ranging from 10° to 55°).

To intuitively analyze the microcrystalline parameter of the samples, the values of d_002_, *L*_*a*_, *L*_*c*_, and the graphitization degree (G) were calculated and listed in Table 3. Compared to raw biochar materials, d_002_ values for all the samples derived from catalytic graphitization were much lower than the value of amorphous carbon (0.3440 nm) and further approached to that of the natural graphite (0.3354 nm)^38^, presenting the development of a good graphitic structure. The trend was also reflected in Fig. S2 where the (002) peaks gradually shifted to 26.55° after the deployment of the catalysts. Besides, the increasing crystallite sizes of *L*_*a*_ and *L*_*c*_ can also be observed, manifesting the growth of graphite microcrystals during graphitization. For these catalytic graphitization cases, synthetic bio-graphite samples derived from hybrid catalysts have smaller d_002_ values than that produced from the single-metal catalyst, suggesting a better graphitic crystallinity. Notably, the G-FeNiMn-111 sample performed the smallest d_002_ value (0.3363 nm), indicating that the graphitic structure in this sample was very close to pure natural graphite. Moreover, the large crystallite sizes of *L*_*a*_ and *L*_*c*_ were achieved in the G-FeNiMn-111 sample. This result demonstrates that the catalyst particles alloyed with Fe, Ni, and Mn play a role in promoting the lateral growth of graphite microcrystals during the heat treatment process and increases the stacking height of graphite microcrystals. The graphitization degrees of the samples are listed in Table 3. In contrast with G-Ni (80.55%), G-FeNi-11 (77.64%), G-FeNi-12 (80.55%), and G-FeNi-21 (83.47%), the G-FeNiMn-111 sample exhibited the most significant graphitization degree of 89.28%. This finding supported the notion that the trimetallic hybrid catalyst could dramatically improve the degree of graphitization.

**Table 3:** Microcrystalline parameters of synthetic bio-graphite samples.

Sample	d_002_ (nm)	G (%)	*L*_*c*_ (nm)	*L*_*a*_ (nm)
G-Ni	0.3371	80.55	15.40	26.93
G-FeNi-11	0.3373	77.64	12.95	37.07
G-FeNi-12	0.3371	80.55	12.55	30.06
G-FeNi-21	0.3368	83.47	12.75	21.84
G-FeNiMn-111	0.3363	89.28	17.74	27.96

### Raman

**Figure 3:**
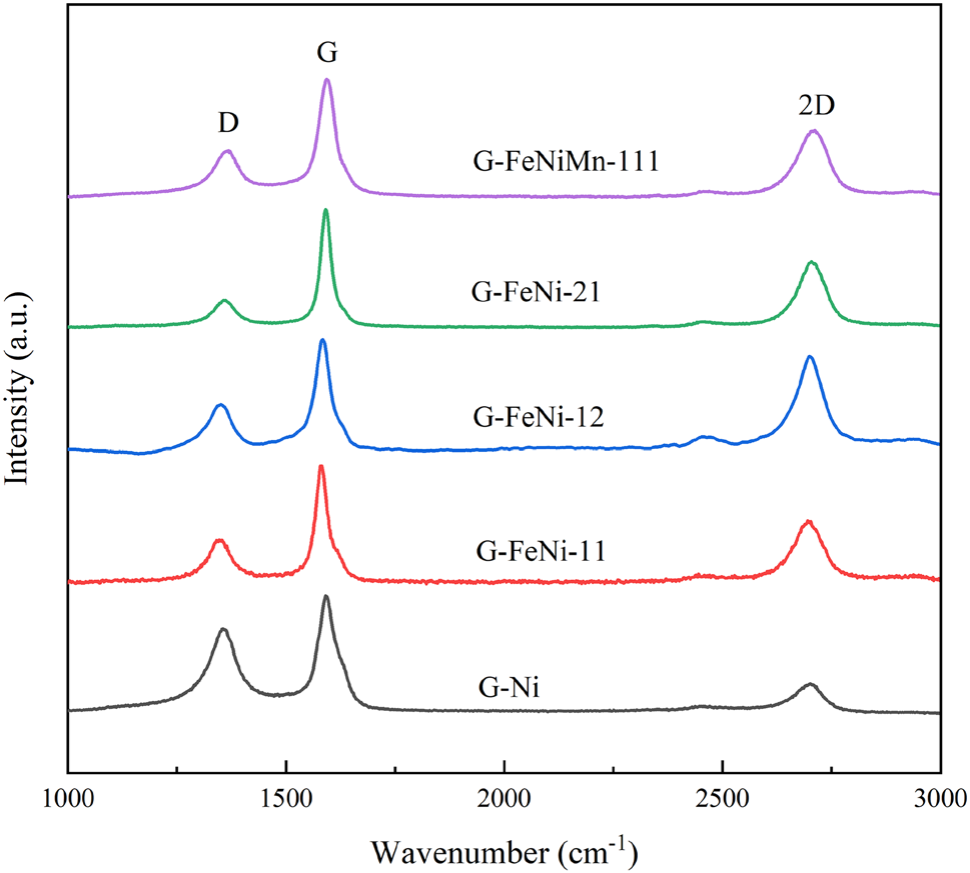
Raman spectrum of synthetic bio-graphite samples (ranging from 1000 to 3000 cm^−1^).

Figure 3 displays the Raman spectrum of synthetic bio-graphite samples, in which three obvious peaks are observed. The peak at about 1360 cm^−1^, which is referred to the D band, corresponds to the disordered carbon^39,40^. The peak at around 1589 cm^−1^, which is termed the G band, is related to the highly ordered graphite^41^. Additionally, the peak at around 2708 cm^−1^, known as the 2D band, is assignable to the excellent regular structure of the graphite as well^42,43^. Differing from the Raman spectrum of the synthetic bio-graphite samples, the Raman spectrum of raw biochar presented in Fig. S3 showed a broad D band, indicating the presence of abundant disordered carbon in the carbon precursor. After loading catalysts, an enhancement of the G peak intensity with respect to the intensity of the D band was observable, corresponding to abundant formation of highly ordered graphite crystallites during the graphitization. Furthermore, the proportion of the graphitic structure after graphitization can reflected by conversion rate (α) which is calculated based on the relative intensities of the D bank and G bank in the Raman spectrum, as shown in Table 4. In this work, the α values of synthetic bio-graphite samples are much higher than that of the raw biochar, indicating a higher degree of graphitization. Compared with the single-metal catalyst, the usage of hybrid catalysts resulted in a higher α value, revealing fewer defects located in graphitic materials. The G-FeNiMn-111 sample, treated by the trimetallic hybrid catalyst, showed α value of 73.95% among the graphitized samples, suggesting a decent catalytic effect during graphitization. The results were in accordance with those of XRD analysis. But the D banks of all the catalytic graphitized sample were still noticeable, which implied that further graphitization of produced bio-graphite samples is still necessary.

**Table 4:** Conversion rates of the synthetic bio-graphite samples.

	G-Ni	G-FeNi-11	G-FeNi-12	G-FeNi-21	G-FeNiMn-111
α	58.32%	73.52%	71.96%	81.23%	73.95%

### TGA

Thermogravimetric analysis (TGA) was then conducted to determine the residual catalyst amounts in synthetic bio-graphite samples after acid leaching. Figure S4 depicted the mass loss of graphite samples (before and after acid leaching) in the thermogravimetric analysis under oxygen ambiance. An obvious plateau can be found after reaching a temperature of 900℃, thereby indicating all graphite has been burnt out and only ash remained inside the crucible. The TGA results showed that, after impregnation and graphitization, the mass loading of the catalyst in the original sample was determined to be approximate 22 wt%. In contrast, after the acid leaching, the amount of residual catalyst fell drastically to approximate 0.7 wt%, revealing most catalyst residuals have been effectively removed from synthetic graphitic materials by using HCl.

### SEM

**Figure 4:**
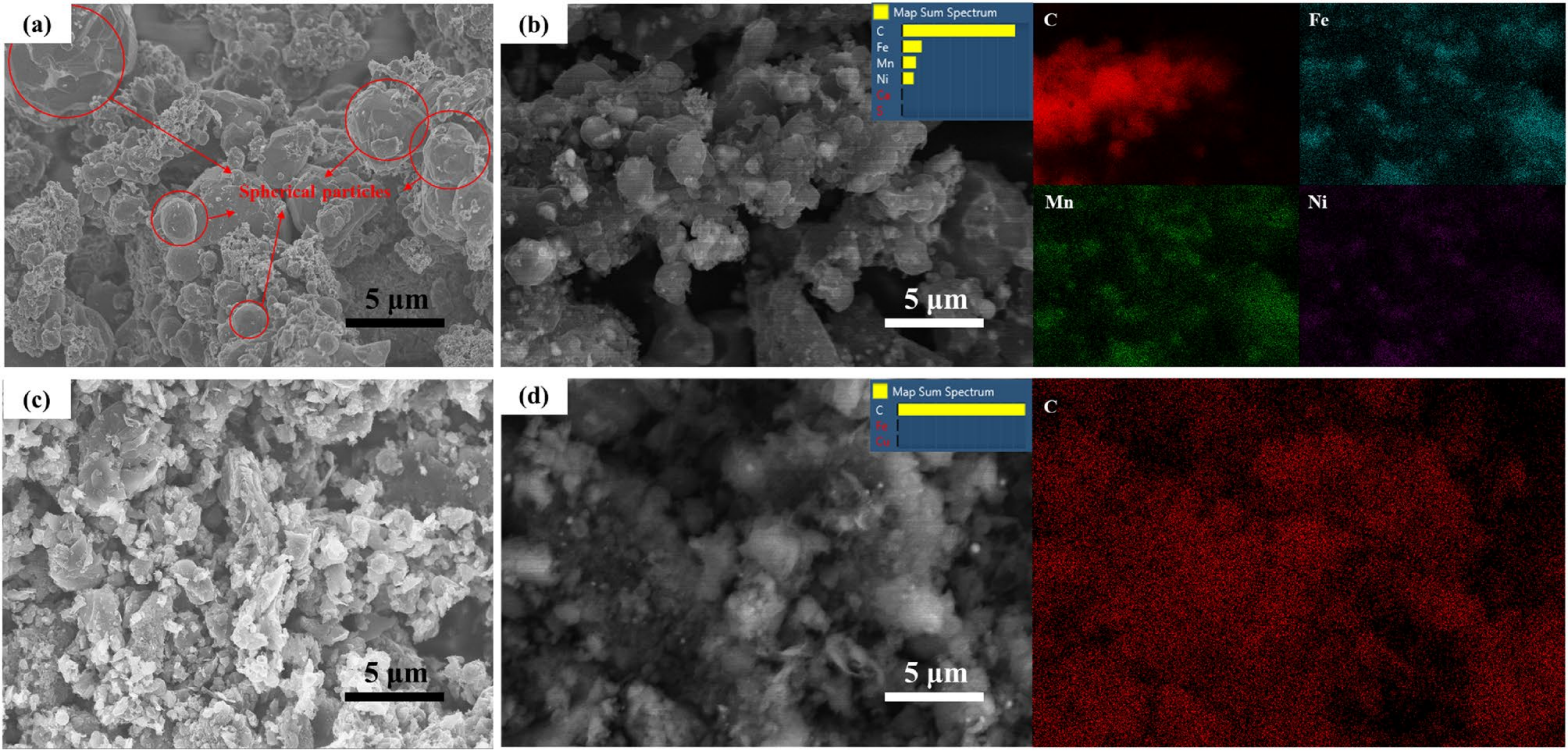
SEM image and EDS mapping analysis of G-FeNiMn-111 sample. (**a**) and (**b**) before acid leaching; (**c**) and (**d**) after acid leaching.

Figure 4a shows SEM images from a back scattered electron signal of the G-FeNiMn-111 sample before acid leaching. The microstructure of the graphitized carbon (before acid leaching) significantly differed from the morphology of the original biochar materials (given in Fig. S5), consisting of unique spherical particles throughout the surface (particles are highlighted by red circles). The EDS analysis in Fig. 4b has confirmed that the solid spherical particles were mainly catalyst particles composed of Fe, Ni, and Mn. In addition to metallic elements, a large amount of carbon was observed in EDS, particularly surrounding catalyst particles, indicating an induced effect of the catalyst on the formation of graphite. However, the mechanism (dissolution–precipitation mechanism or formation-decomposition mechanism) that dominated in this process cannot be determined. Figure 4c showed the SEM images of the G-FeNiMn-111 sample after acid leaching. Upon comparison between Fig. 4a and c, it turned out that all the alloy particles vanished after being treated with acid and no metallic elements were detected in its EDS analysis (Fig. 4d), which further supports that all metals were dissolved by hydrochloric acid, leaving only the graphite behind. The results are in accordance with TGA results, which indicate a low content of catalyst residuals in synthetic bio-graphite samples.

### HRTEM

**Figure 5:**
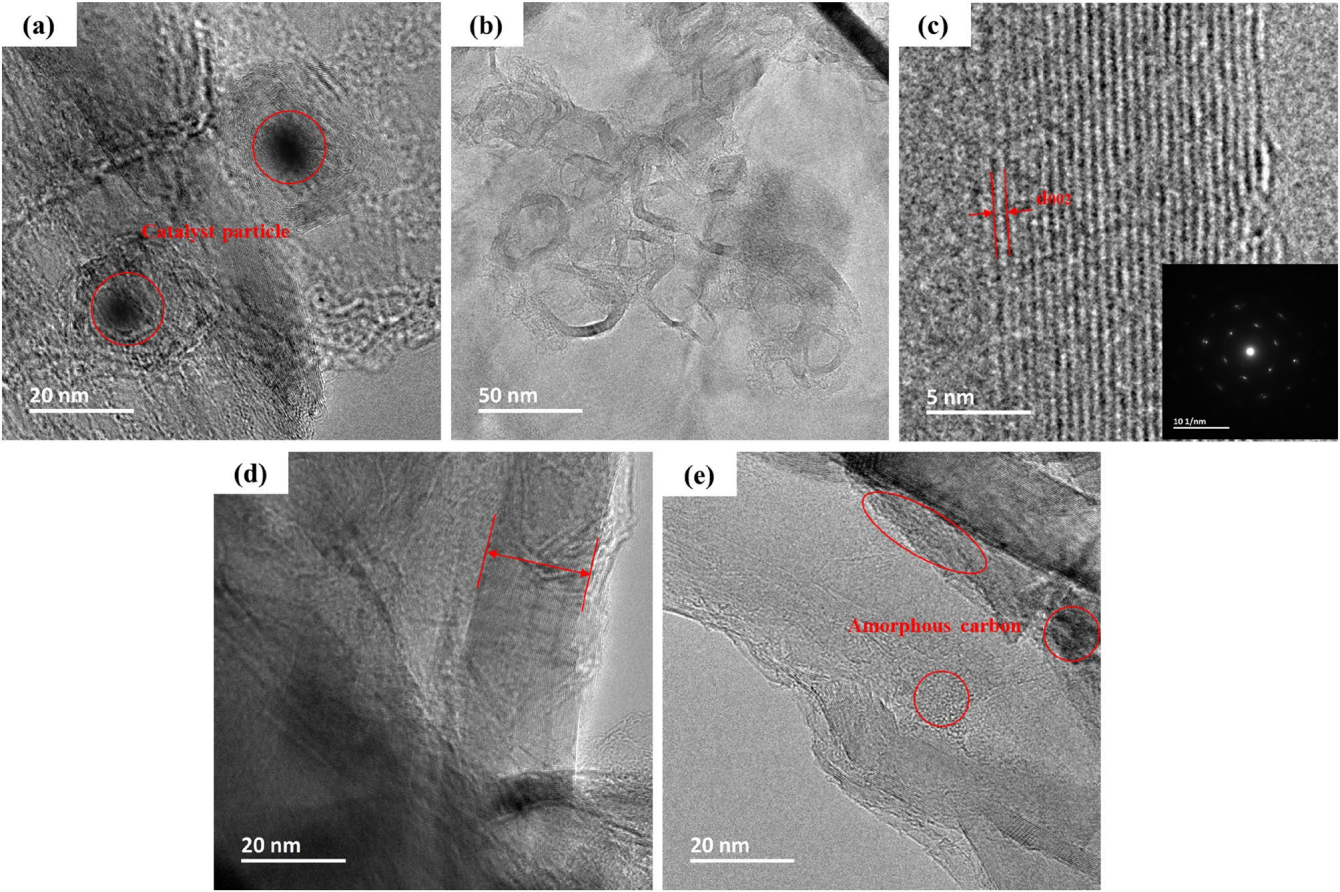
HRTEM image of G-FeNiMn-111 sample. (**a**) before acid leaching; (**b**–**e**) after acid leaching.

The crystallinity and microstructure of the synthetic bio-graphite were analyzed by using HRTEM. According to the HRTEM micrographs of the G-FeNiMn-111 sample shown in Fig. 5a, a typical onion-like microstructure was observed, featuring concentric catalyst particles embedded and surrounded by multiple overlapping curved and ordered graphitic shells. The concentric particles verified that, during heating, the catalyst particles moved through the amorphous carbon areas which subsequently precipitated as graphitic ordered regions. This result aligns well with the literature references that proposed the mechanism of catalytic graphitization^26,34,44,45^. During the graphitization, the carbon atoms resulting from the decomposition of the biochar matrix dissolved into the liquid-state catalyst particles and precipitated as graphene sheets on their surface. The nucleation and growth of carbon species during the process resulted in the encapsulation of metal particles by single- or multi-graphitic sheets^46^.

Figure 5b shows the hollow microstructure observed in synthetic graphitic materials after acid etching, manifesting that catalyst particles are almost completely dissolved and removed from the synthetic bio-graphite. Figure 5c is the magnified image of highly ordered graphitic crystallites, in which an interlayer distance below 0.34 nm was found on the lattice fringes of the surrounding stacked structure. Additionally, the d_002_ value was calculated based on selected area electron diffraction (SAED) patterns of the G-FeNiMn-111 sample, and a same d_002_ value was obtained as the result derived from XRD, further inferring the high crystallinity. Figure 5d shows highly crystalline graphitic domains, creating long-range stacked carbon layers, which verified the large crystallite sizes *L*_*a*_ and *L*_*c*_ obtained from XRD. However, some less ordered turbostratic regions with higher interlayer distances were also found in Fig. 5e. These results were consistent Raman spectrum where the presence of the D band was found, implying more studies should be conducted to convert the remaining amorphous carbon into a highly ordered sheet-like graphitized structure. And one possible way could be increasing the catalyst loading amount during the graphitization process.

### BET specific surface area

The BET surface areas of the synthetic bio-graphite samples are listed in Table 5, ranging from about 42 to 93 m^2^/g, significantly lower than that of biochar (324 m^2^/g). The result indicates a significant pore closure effect caused by the combined thermal treatment and the addition of catalysts. However, these values are still much higher than that of commercial graphite (5–20 m^2^/g^47^). The dissolution of the metallic catalyst during acid leaching might cause an increase in the surface area owing to the generation of new pores. Therefore, further thermal treatment of bio-graphite samples is highly suggested to destroy the pores generated by acid leaching for further research. It has been confirmed that irreversible capacity can be correlated to the graphite’s BET specific surface area, giving an almost linear relationship^47,48^. In this work, a relatively high BET surface area was observed in the G-FeNiMn-111 sample (93 m^2^/g), which could have a negative influence on its electrochemical performance.

**Table 5:** BET surface areas of original biochar and the synthetic bio-graphite samples.

	Biochar	G-Ni	G-FeNi-11	G-FeNi-12	G-FeNi-21	G-FeNiMn-111
BET surface area (m^2^/g)	324	67	88	69	42	93

### Electrochemical characterization

**Figure 6:**
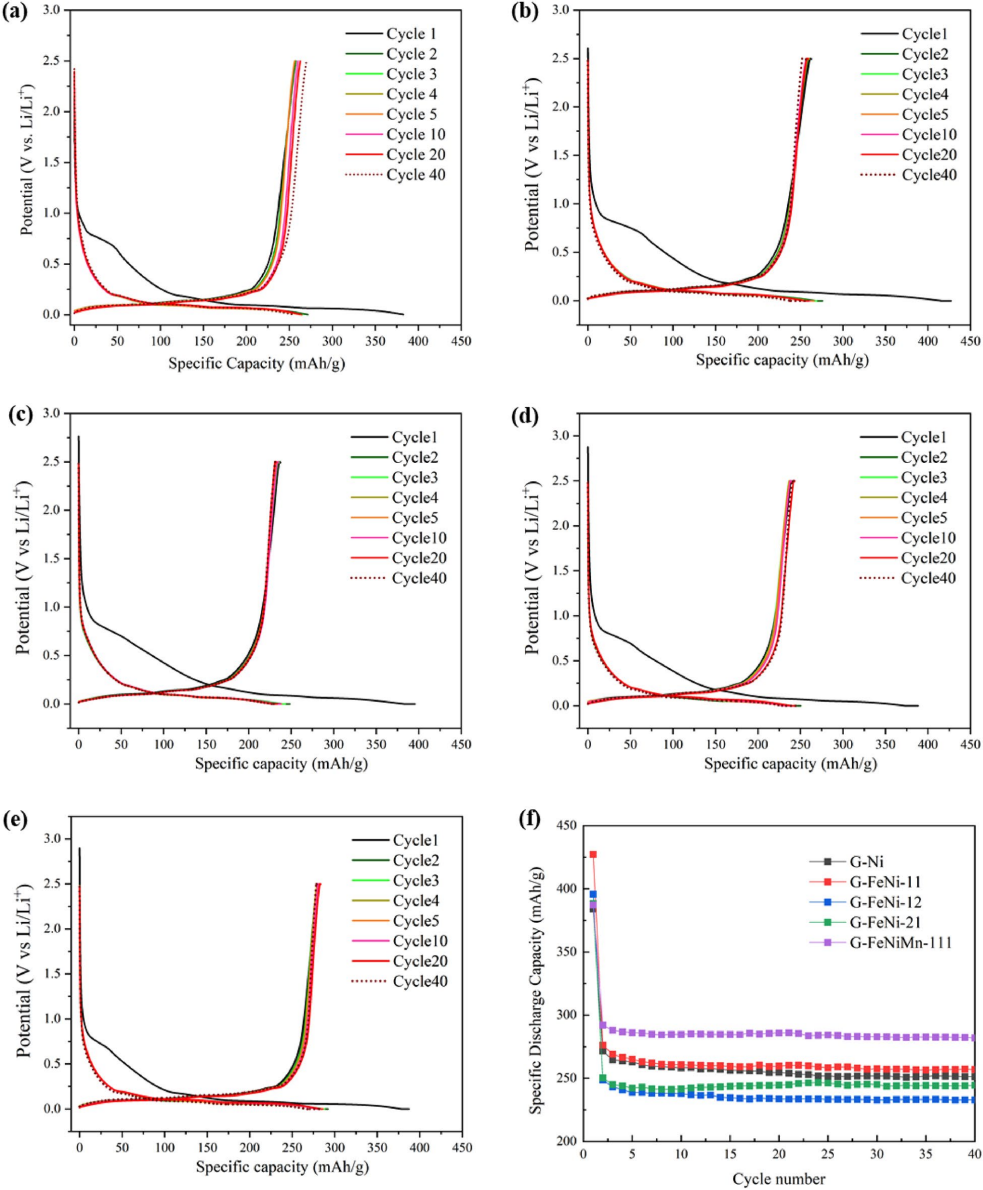
The galvanostatic charge/discharge cycles of all the synthetic bio-graphite samples at 20 mA/h. (**a**) G-Ni; (**b**) G-FeNi-11; (**c**) G-FeNi-12; (**d**) G-FeNi-21; (**e**) G-FeNiMn-111 and (**f**) the cycling performance (40 cycles) of all bio-graphite samples at 20 mA/g.

**Figure 7:**
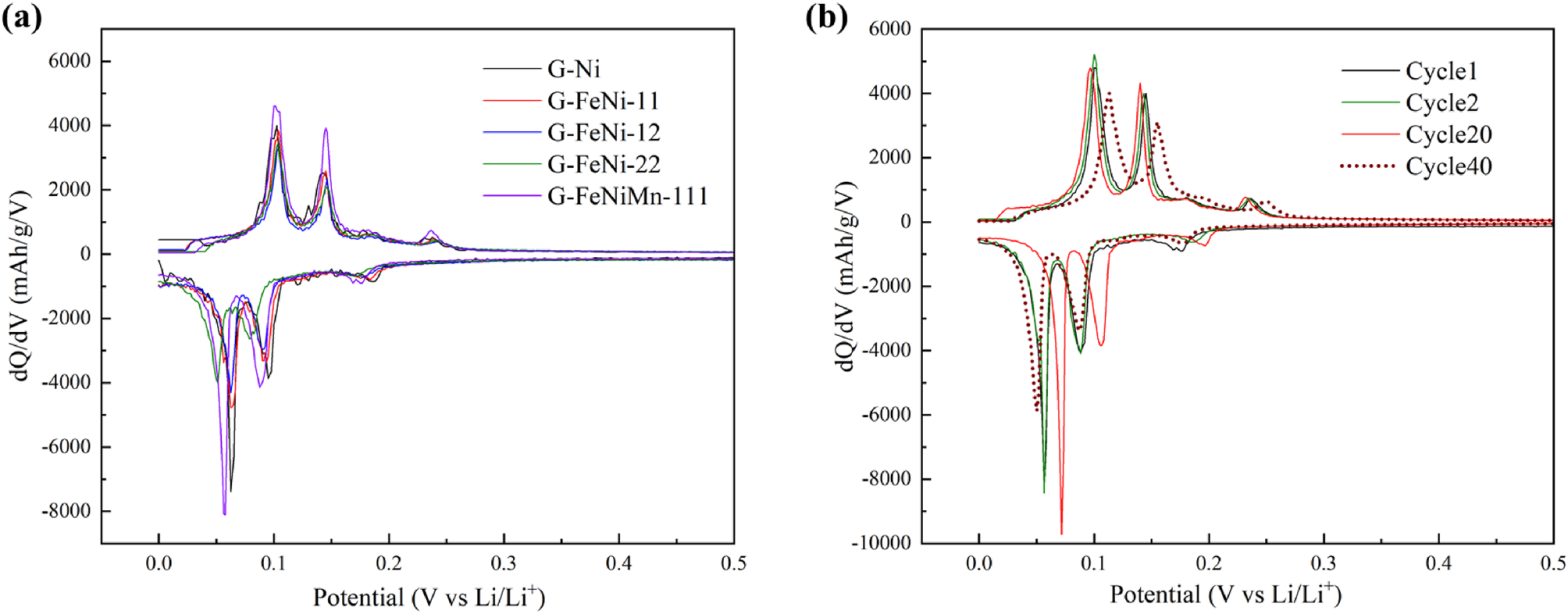
Differential capacity (dQ/dV vs Potential) curves. (**a**) all the synthetic bio-graphite samples at 1st cycle and (**b**) G-FeNiMn-111 at 1st, 2nd, 20th and 40th cycles.

Figure 6 summarizes the study on the electrochemical performance of synthetic bio-graphite samples as negative electrodes in lithium half-cells. The electrodes were cycledbetween 0 and 3.0 V Li^+^/Li at a current of 20 mA/g for which the charge and discharge curves are provided in Fig. 6a–e. For all samples, a broad plateau was observed around 0.71 V in the first discharge curve, indicating the formation of a solid electrolyte interphase (SEI) layer^49^. Other plateaus appeared between 0.21 and 0.084 V, usually associated to the Li^+^ intercalation into the graphite layers^50^. Such an observation indicates the graphitic structure of the samples. Similar observation is also reflected in the differential capacity plots (see Fig. 7) generated from the galvanostatic curves. The peaks at various potentials are reminiscent of the staging mechanism of ion intercalation typically observed in graphite intercalation compounds^51^. Figure 6f indicates that the initial discharge capacities of G-Fe, G-FeNi-11, G-FeNi-12, G-FeNi-21, and G-FeNiMn-111 are 383.9, 427.23, 395.73, 388.30, and 387.24 mAh g^−1^, respectively, while the corresponding charge capacities are 255.50, 262.56, 237.17, 240.08, and 292.57 mAh g^−1^, respectively. As shown in Fig. S6, the corresponding coulombic efficiencies are as high as 66.55%, 61.45%, 59.93%, 61.82%, and 75.55%, respectively. Compared with other synthetic bio-graphite samples derived from single-metal and hybrid catalysts, G-FeNiMn-111 exhibited the highest reversible capacity (293 mAh g^−1^) with a columbic efficiency of 75.55%. The improvement of reversible capacity was owing to the better-ordered graphite sheet layers in G-FeNiMn-111, which could facilitate the intercalation and deintercalation of Li-ions and improve fast ion diffusion and transportation^52^.

However, the reversible capacity and initial coulombic efficiency were still low and not comparable to those of commercial graphite products. An possible explanation for the result was that the unconverted amorphous carbon in graphite samples led to the reductive decomposition of the electrolyte, resulting in the thick SEI formation before stabilizing the electrode–electrolyte interface, eventually offering a lower reversible capacity than that of commercial^53^. Therefore, more studies are recommended to decrease the porosity and surface area of graphite materials, further approaching commercial graphite products (355 mAh g^−1^). A feasible solutions could be an additional heat treatment to eliminate the pores in the carbon materials, which has been verified by literature^25^.

**Figure 8:**
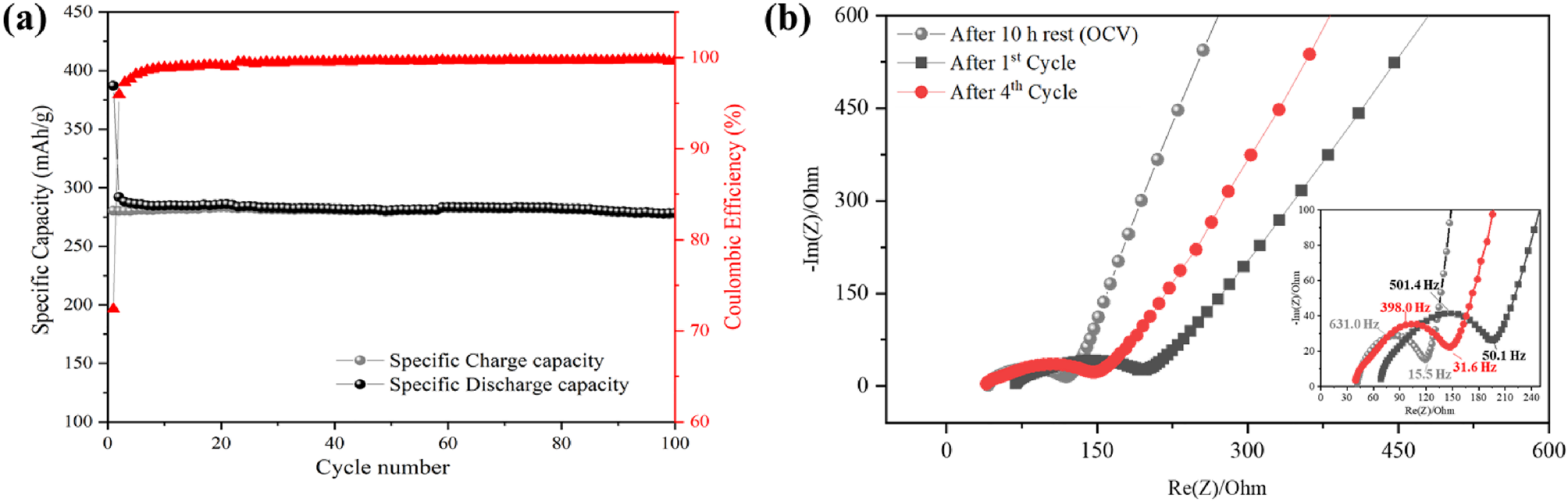
(**a**) The long-term cycling performance (100 cycles) of G-FeNiMn-111 sample at 20 mA/g. (**b**) Nyquist plots (at OCV, after 1st cycle and after 4th cycle) of G-FeNiMn-111 graphite sample in Li-ion half-cell in 2-electrode configuration.

The long-term cycling performance of G-FeNiMn-111 (the sample with best performance) was tested at 20 mA/g for 100 cycles and shown in Fig. 8a. It turned out that G-FeNiMn-111 exhibited a high capacity of 292.57 mAh g^−1^ in the initial cycle and retains a capacity of 279 mAh g^−1^ after 100 cycles. The relevant retention could reach as high as 98.73%, reflecting the excellent stability and cycling ability of G-FeNiMn-111. Moreover, the coulombic efficiency of G-FeNiMn-111 maintained in a stable range between 95 and 100% during the whole cycling performance. The good electrochemical properties were ascribed to its highly ordered graphitic structure that was induced by loading Fe, Ni, and Mn as catalysts in the graphitization process. The highly ordered layered structure could facilitate the migration of Li-ions and electron transfer^52^, enhancing the lithium-ion embedding capacity and thus boosting the electrochemical performance of the graphite in LIBs. Even yet, the progressive rate test and long cycle life at high current (Figs. S7 and S8, respectively) demonstrated the need for electrode engineering and further research into graphite in various electrolytes. The best performing G-FeNiMn-111 electrode was further tested using EIS for selected cycles (Fig. S9) to understand the electronic, ionic and interfacial properties at different OCV and after cycling. The observed EIS response can be attributed to the two electrodes and the electrode–electrolyte interfaces. The Nyquist plots in Fig. 8b show a combination of semi-circles and diffusion arcs. After cycling, the size of the semi-circles increases clearly showing an increase in the charge transfer resistance as a result of solid electrolyte interphase layers formed on the graphite and lithium metal electrodes^54,55^.

## Conclusion

The study successfully prepared battery-use synthetic bio-graphite samples by catalytic graphitization of biochar using hybrid catalysts at 1300 °C. The influence of the hybrid catalysts on the graphitic structure and electrochemical properties of the resulting bio-graphite were studied. Specifically, bimetallic hybrid catalysts (Ni and Fe with ratios of 1:1, 1:2, and 2:1) and a trimetallic hybrid catalyst (Ni, Fe, and Mn with a ratio of 1:1:1) were deployed. Results indicated that the catalyst with three metals (Ni, Fe, and Mn with a ratio of 1:1:1) was the most effective catalyst for graphitizing the biochar precursor The alloy nanoparticles and the unpaired electrons in Ni, Mn and Fe may contribute to the effective improvement. Electrochemical performance test showed that bio-graphite sample produced via the trimetallic hybrid catalyst displayed better electrochemical performances as anode materials than the other samples, e.g., good reversible capacity (293 mAh g^−1^ at 20 mA/g) and stable cycle performance (capacity retention over than 98% after 100 cycles). The synergistic effect caused by the use of different metals was proved to be effective for catalytic graphitization in terms of graphite crystalline and the corresponding electrochemical performance. However, there is still a gap between bio-graphite and commercial graphite. Future works are recommended to determine the mechanism of benefit underlying the benefits of the trimetallic hybrid catalyst and emphasize exploring various methods (e.g., introducing a heat treatment to bio-graphite materials or increasing the catalyst loading amount) to improve the degree of graphitization, conversion rate and electrochemical performance of bio-graphite.

### Supplementary Information

Supplementary Figures.

## Data Availability

The datasets used and/or analyzed during the current study available from the corresponding author on reasonable request.
