# Supplementary material for: Bio-based anode material production for lithium–ion batteries through catalytic graphitization of biochar: the deployment of hybrid catalysts
Source: Sci Rep. 2024 Feb 17;14:3966. doi: 10.1038/s41598-024-54509-8 (PMC10874404; doi:10.1038/s41598-024-54509-8)
Supplement: Supplementary file 1 — Supplementary Figures. [file 41598_2024_54509_MOESM1_ESM.docx]

**Bio-based anode material production for lithium-ion batteries through catalytic graphitization of biochar: the deployment of hybrid catalysts**

Ziyi Shi^a^, Yanghao Jin^a^, Tong Han^a,^*, Hanmin Yang^a^, Ritambhara Gond^b^, Yaprak Subasi^b^, Habtom Desta Asfaw^b^, Reza Younesi^b^, Pär G. Jönsson^a^ , Weihong Yang^a^

^a^ Department of Material Science and Engineering, KTH Royal Institute of Technology, Stockholm, 114 28, Sweden

^b^ Department of Chemistry - Ångström Laboratory, Uppsala University, Lägerhyddsvägen 1, Box 538, Uppsala 75121, Sweden

* Correspondence: [tongh@kth.se](mailto:tongh@kth.se)

# **Figures**


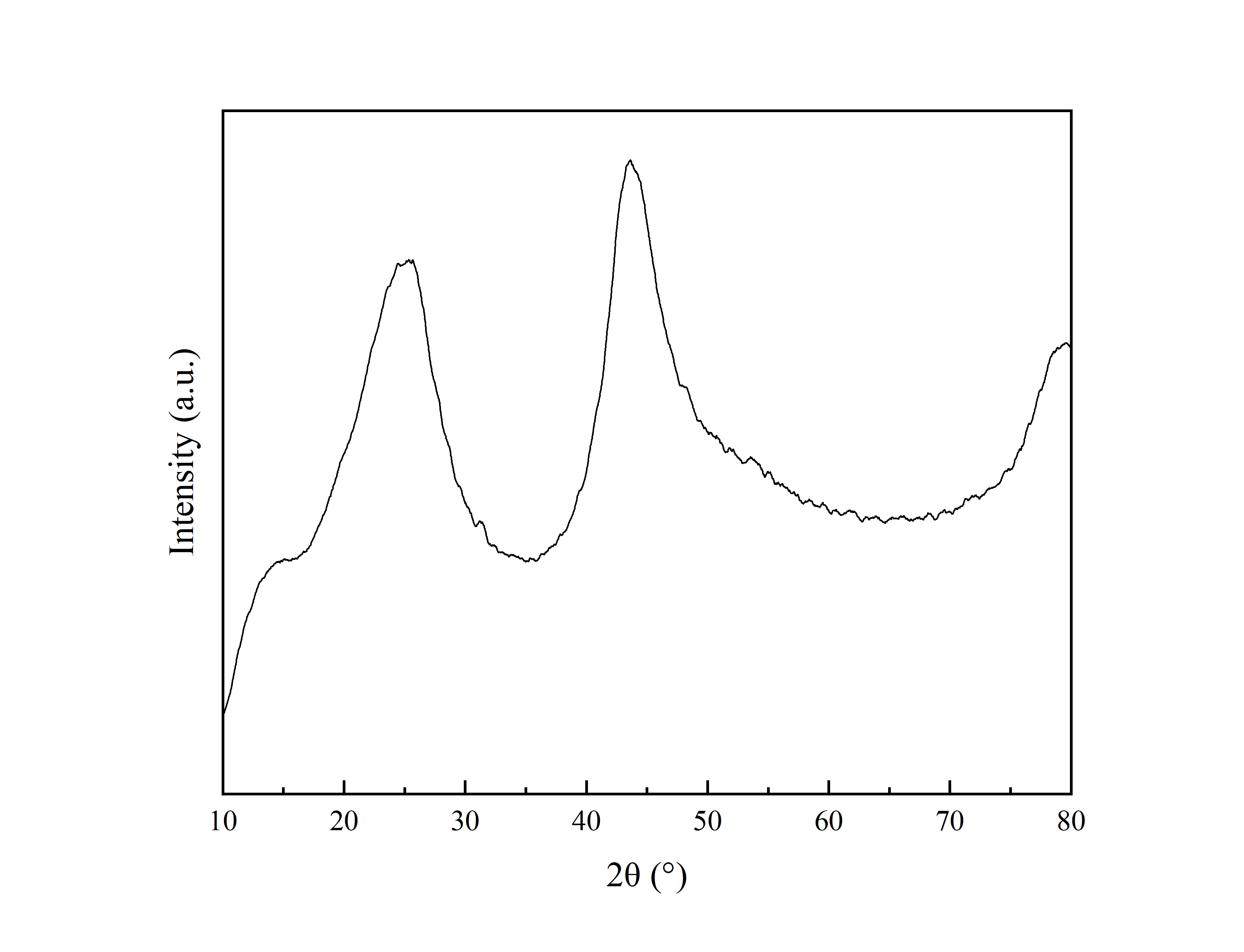


Figure S1. XRD pattern of biochar.


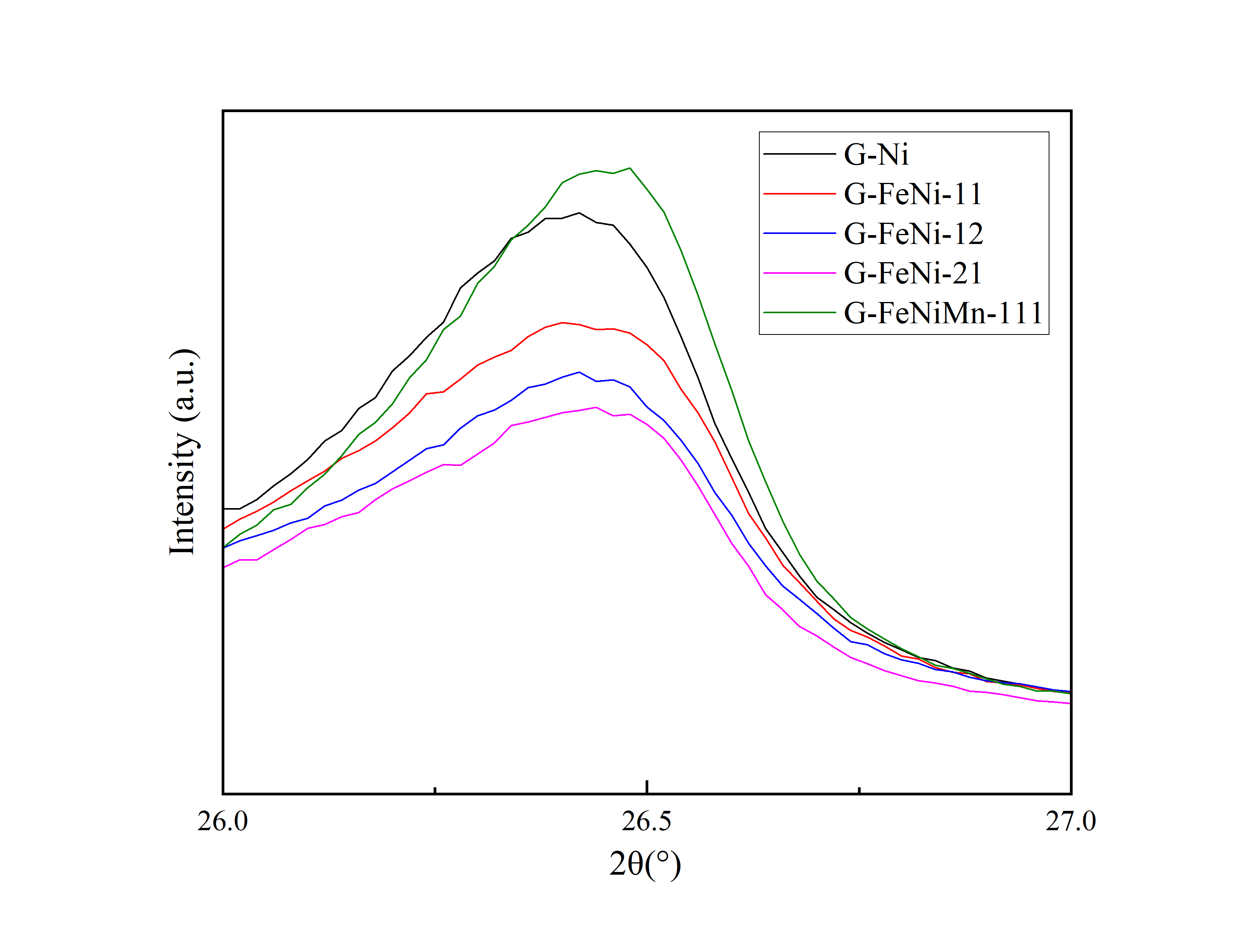


**Figure S2**. The shift of (002) peaks of synthetic bio-graphite samples.


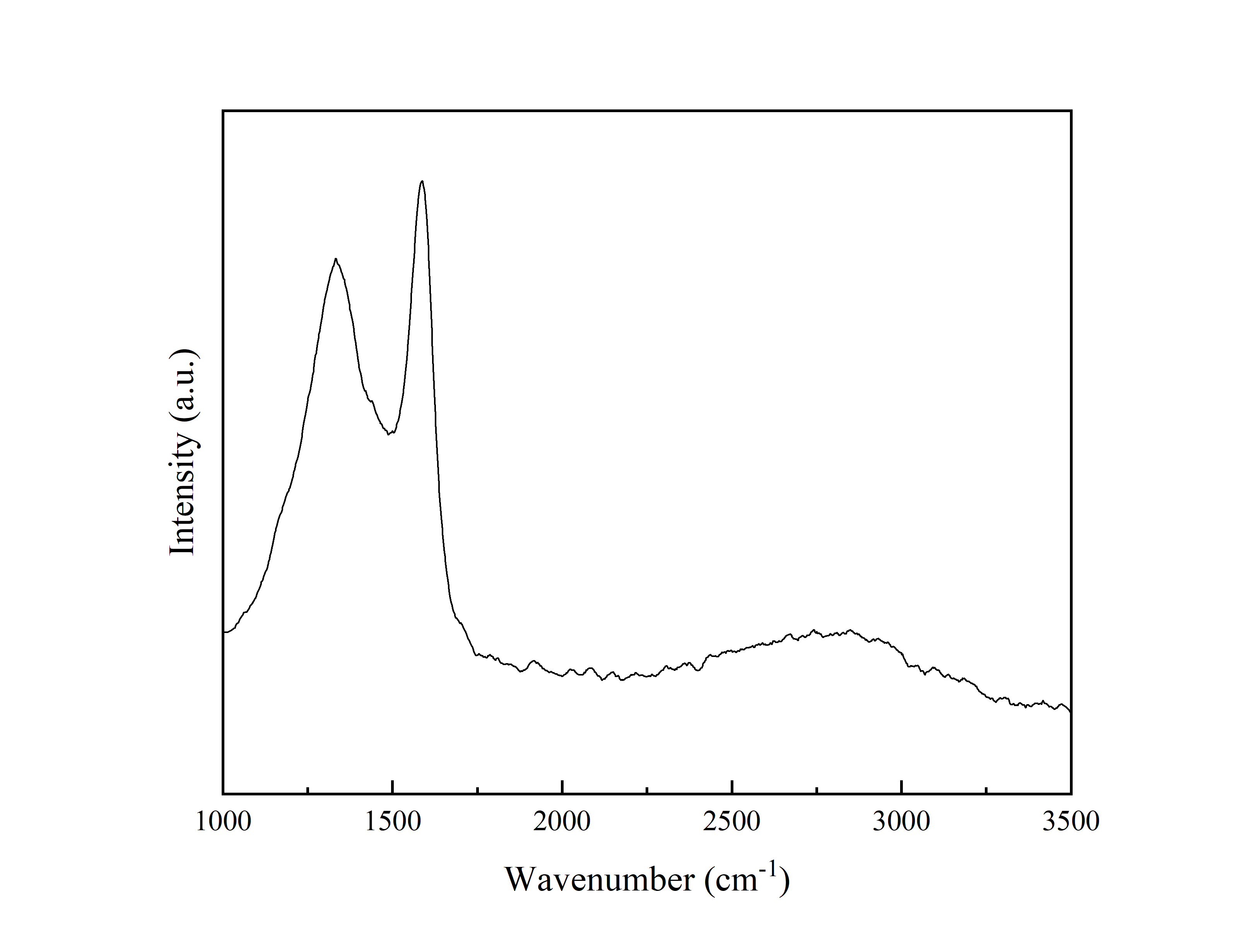


Figure S3. Raman spectrum of biochar.


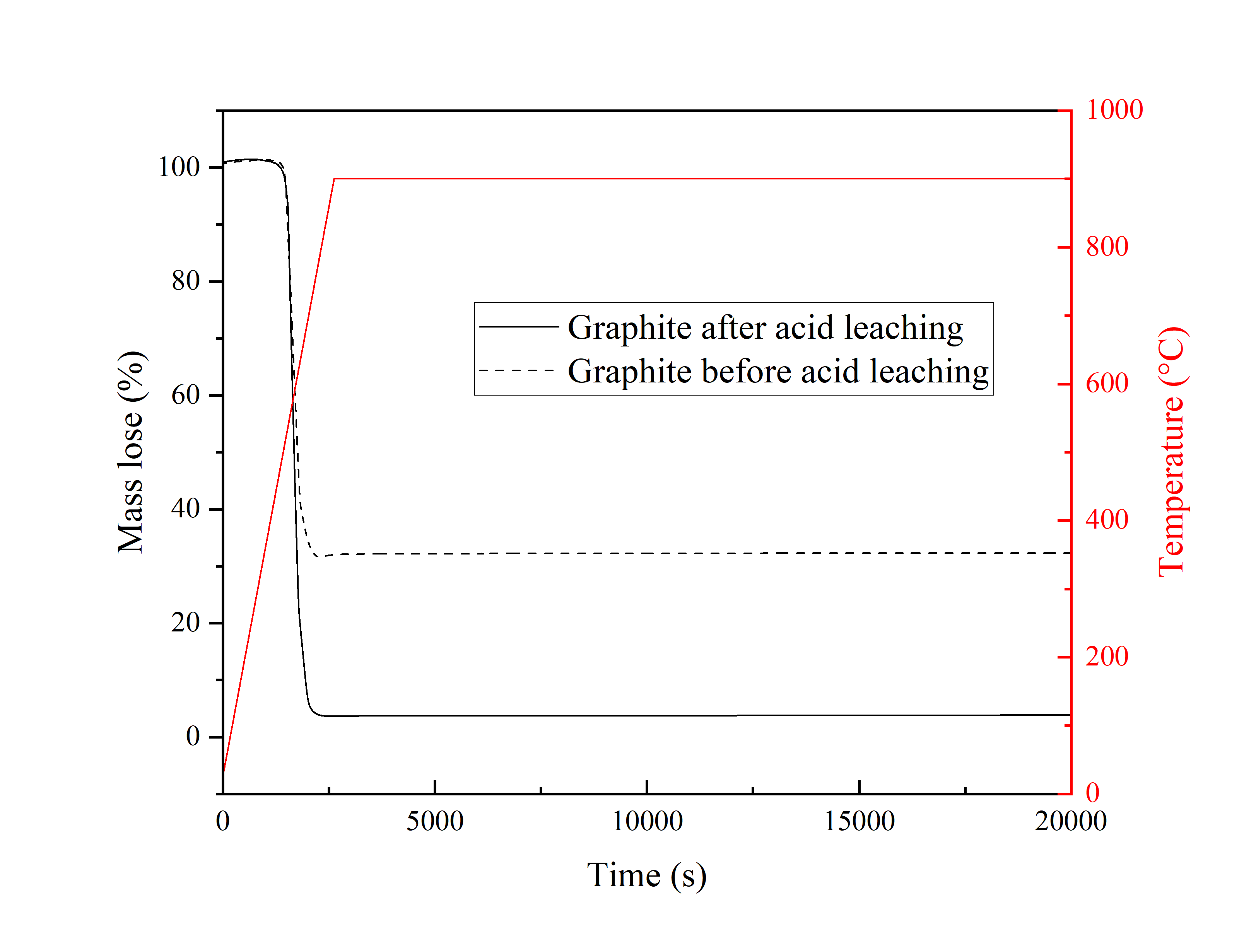


Figure S4. TGA of graphite sample before and after acid leaching.


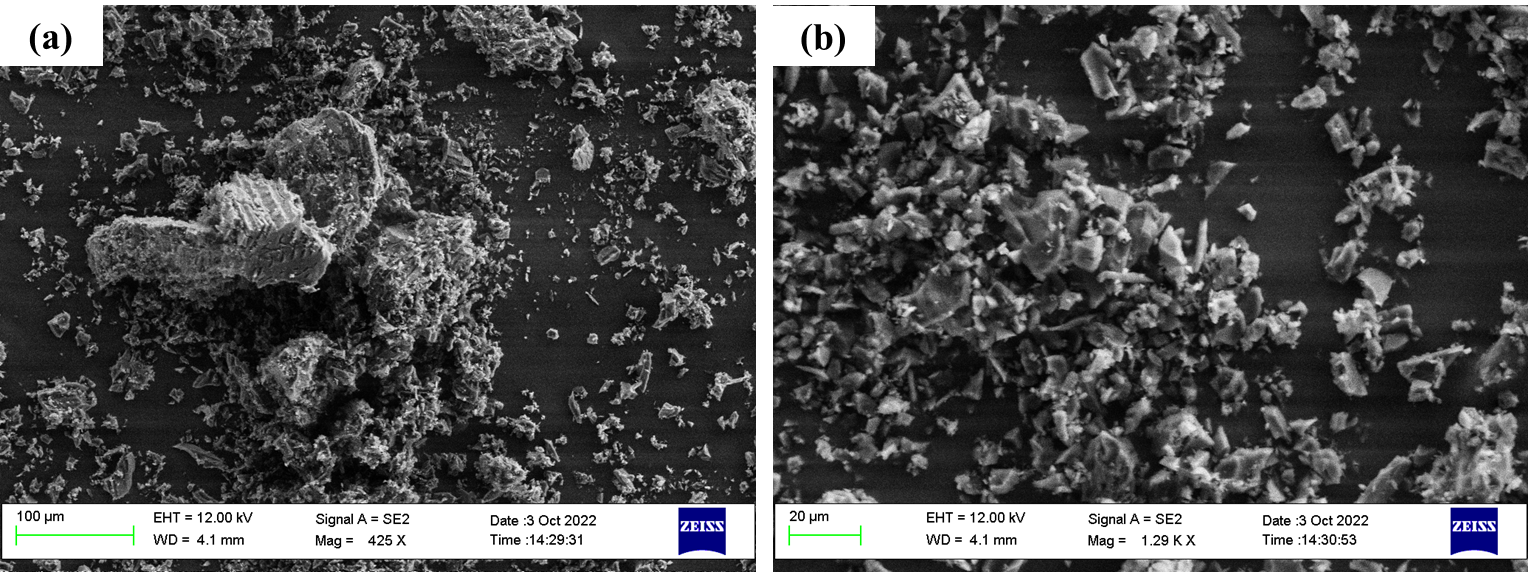


Figure S5. SEM of biochar. (a) Magnification: 425X and (b) Magnification: 1.29kX


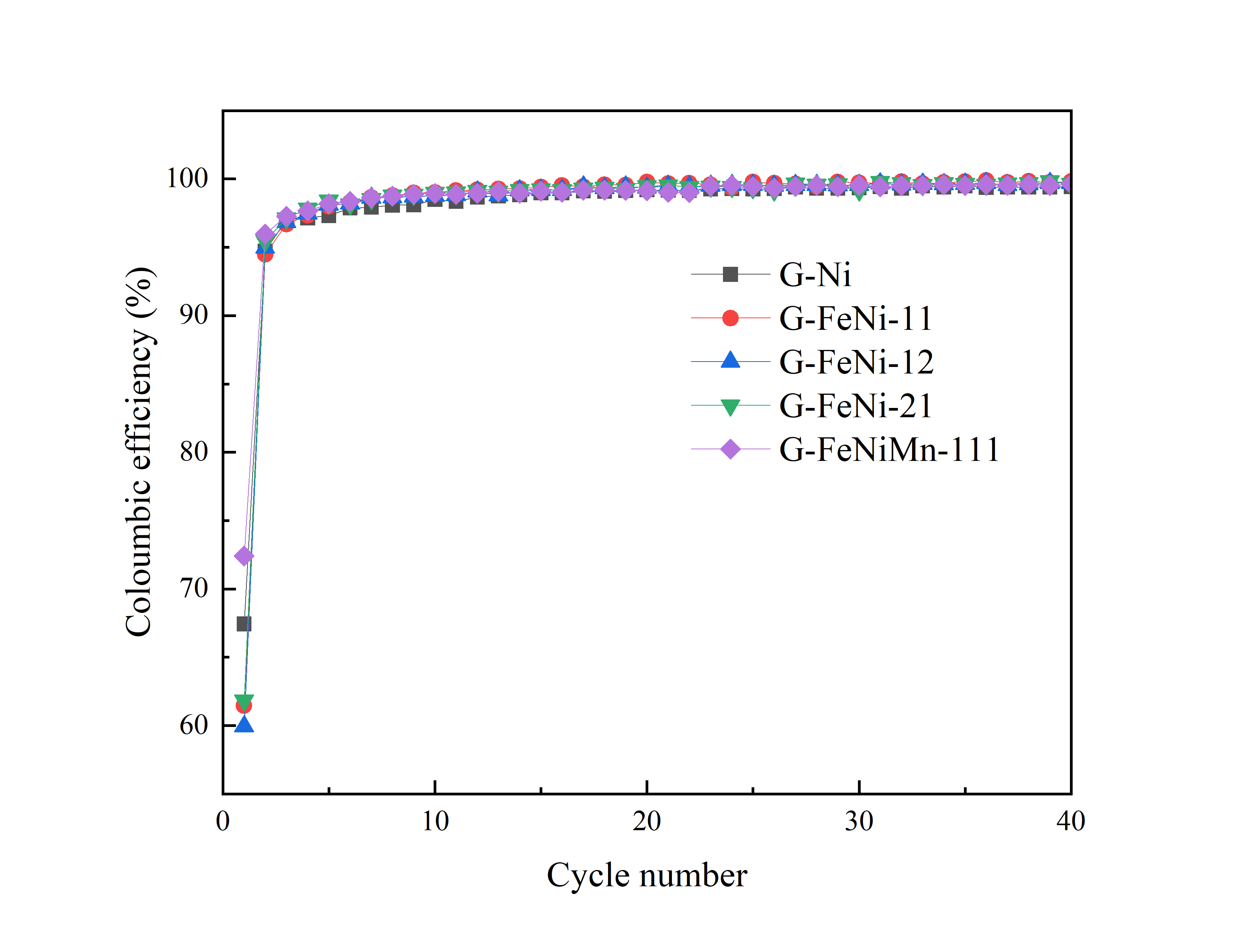


Figure S6. The coloumbic efficiency of all the bio-graphite samples.

Figure S7. The progressive rate test of G-FeNiMn-111 in Li half-cell in LP57 electrolyte.

Figure S8. The specific capacity, coloumbic efficiency vs cycle number results at very high current followed by 3 formation cycles at 0.1C for the best performing G-FeNiMn-111 sample.

Figure S9. The galvanostatic charge/discharge cycles of G-FeNiMn-111 sample, employed to perform PEIS test shown in Figure 8(b) in the main manuscript.
